# Cardiovascular mortality in peritoneal dialysis: the impact of mineral disorders

**DOI:** 10.1590/2175-8239-JBN-2020-0040

**Published:** 2021-02-08

**Authors:** César Truyts, Melani Custodio, Roberto Pecoit-Filho, Thyago Proenca de Moraes, Vanda Jorgetti

**Affiliations:** 1 Universidade de São Paulo, Laboratório de Fisiopatologia Renal, São Paulo, SP, Brasil.; 2 Pontifícia Universidade Católica do Paraná, Faculdade de Medicina, Curitiba, PR, Brasil.

**Keywords:** Phosphates, Renal Insufficiency, Chronic, Mortality, Peritoneal Dialysis, Fosfatos, Insuficiência Renal Crônica, Mortalidade, Diálise Peritoneal

## Abstract

**Introduction:**

Mineral and bone disorders (MBD) are associated with higher mortality in dialysis patients. The main guidelines related to the subject, Kidney Disease Outcomes Quality Initiative (KDOQI) and Kidney Disease: Improving Global Outcomes (KDIGO), were elaborated based on published information from hemodialysis participants. The aim of our study was to evaluate the impact of calcium (Ca), phosphorus (P), and parathyroid hormone (PTH) (according to guideline ranges from KDOQI and KDIGO) on the cardiovascular mortality of peritoneal dialysis (PD) patients.

**Methods:**

We used the BRAZPDII database, an observational multi-centric prospective study, which assessed participants on PD between December 2004 and January 2011. Amongst 9,905 participants included in this database, we analyzed 4424 participants who were on PD for at least 6 months. The appropriate confounding variables were entered into the model. Serum levels of Ca, P, and PTH were the variables of interest for the purposes of the current study.

**Results:**

We found a significant association between high P serum levels, categorized by KDOQI and KDIGO (P above 5.5 mg/dL), and cardiovascular survival (*p* < 0.01). Likewise, a compelling association was found between lower levels of PTH, categorized by guidelines (KDOQI and KDIGO - PTH less than 150 pg/mL, *p* < 0.01), and cardiovascular survival.

**Conclusion:**

In conclusion, levels of P above and PTH below the values proposed by KDOQI and KDIGO were associated with cardiovascular mortality in PD patients.

## Resumo

**Introdução:**

Os distúrbios minerais e ósseos (DMO) estão associados a maior mortalidade em pacientes de diálise. As principais diretrizes relacionadas ao assunto, *Kidney Disease Outcomes Quality Initiative* (KDOQI) e *Kidney Disease: Improving Global Outcomes* (KDIGO) foram elaboradas com base em informações publicadas de pacientes em hemodiálise. O objetivo do nosso estudo foi avaliar o impacto do cálcio (Ca), fósforo (P) e paratormônio (PTH) (de acordo com as faixas propostas pelas diretrizes do KDOQI e KDIGO) na mortalidade cardiovascular de pacientes em diálise peritoneal (DP).

**Métodos:**

Utilizamos o banco de dados BRAZPDII, um estudo prospectivo observacional multicêntrico, que avaliou participantes de DP entre dezembro de 2004 e janeiro de 2011. Entre os 9.905 participantes incluídos neste banco de dados, analisamos 4.424 que estavam em DP há pelo menos 6 meses. As variáveis de confusão apropriadas foram inseridas no modelo. Os níveis séricos de Ca, P e PTH foram as variáveis de interesse para os fins do presente estudo.

**Resultados:**

Encontramos uma associação significativa entre níveis séricos de P elevados, categorizados por KDOQI e KDIGO (P acima de 5,5 mg/dL), e sobrevivência cardiovascular (*p* < 0,01). Da mesma forma, foi encontrada uma associação convincente entre níveis mais baixos de PTH, categorizados por diretrizes (KDOQI e KDIGO - PTH inferior a 150 pg/mL, *p* < 0,01), e sobrevivência cardiovascular.

**Conclusão:**

Em conclusão, níveis de P acima e PTH abaixo dos valores propostos por KDOQI e KDIGO foram associados à mortalidade cardiovascular em pacientes de DP.

## Introduction

Peritoneal dialysis (PD) is an important alternative for the treatment of patients with chronic kidney disease (CKD), with potential advantages from a clinical, logistical, and cost perspectives in relation to hemodialysis (HD)^1^. Cardiovascular disease accounts for approximately half of the cases of death and one third of the hospitalizations of these patients^2^. Mineral and bone disorders (MBD) including abnormalities of serum calcium (Ca), phosphorus (P), parathyroid hormone (PTH), vitamin D, and fibroblast growth factor 23 (FGF-23), and also abnormalities in bone turnover and extra-osseous calcifications contribute to the morbidity and to poor outcomes in these patients^3^^-^ 5.

Several registries and cohort studies in the dialysis population have contributed to the development of the Kidney Disease Outcomes Quality Initiative (KDOQI) and Kidney Disease: Improving Global Outcomes (KDIGO) guidelines for the management of MBD^6^^-^ 11. These guidelines are vastly based on studies focused on the HD population. The purpose of this research was to determine the relationship between the values recommended by these guidelines for Ca, P, and PTH, and cardiovascular mortality.

## Patients and methods

The Brazilian Peritoneal Dialysis Multicenter Study II (BRAZPD II) is a national study involving a representative sample of PD participants, which collected information between December 2004 and January 2011. This database contains demographic, clinical, and laboratory information including the routine monitoring of MBD of 9,905 participants on PD from 122 centers across the country. The data were entered by each center using PDnet^®^ platform. Based on these characteristics, BRAZPD II represents a good opportunity to analyze the impact of the current recommendation for targets related to MBD on clinical outcomes of PD participants^12^. Briefly, after being selected to participate in the study, each clinic submitted the project to the local ethic committee, and all patients signed an informed consent.

We enrolled all 4,424 incident patients on PD for at least 6 months. The endpoint event was death from all causes, and then death from cardiovascular causes (coronary disease and cardiac failure). Causes of censoring were transfer to hemodialysis or kidney transplantation or renal function recovery or transfer to another dialysis center.

### Statistical analysis

The results are expressed in mean or median with standard deviation or interquartile, according to Shapiro-Wilk test.

Cox and competing risk models were used to evaluate the strength of the associations between Ca, P, PTH, and death (from all causes and death from cardiovascular causes). The following variables were included in this analysis: age, diabetes mellitus (DM), coronary artery disease (CAD), residual diuresis (RD - presence and absence), and albumin. The variables of interest were Ca, P, and PTH.

Graphs were developed to compare the groups for phosphorus and PTH, categorized by the guidelines, calculated using a Cox proportional hazard regression model.

The statistical test used was log-rank test. Statistical significance was defined as *p* < 0.05. We used R-project software version 3.5.2 for analyses.

## Results

Demographic and clinical characteristics, risk factors, and results of the biochemical analysis of participants included in the study are described in Table 1. A total of 4,424 patients were analyzed, the average age was 59 years, and other demographic information are as follows: half (52%) of the patients (n = 2300) were female, 62.8% were white, more than 75% of patients were hypertensive, 55% were diabetic, 21.8% of patients had CAD, average BMI was 24 kg/m^2^ and 70% had RD. Median serum Ca was 9.5 mg/dL, P was 4.8 mg/dL, PTH 256 pg/mL, and albumin 3.4 mg/dL. Maximum follow up was 72 months and median follow up was 17 months.

**Table 1:** Baseline patient characteristics

n	4424
Demographic parameters	
Age, years	59.8 [49.2-70.6]
Gender (female), %	52.9
Race (Caucasian), %	62.8
Hypertension (no), %	23.4
Diabetes (yes), %	55.3
CAD (yes),%	21.8
BMI, Kg/m^2^	24.1 [21.7-27.3]
Residual diuresis (no), %	30.1
PD modality (APD), %	45.6
Calcium dialysate (2.5 mEq/L), %	8.6
**Laboratory parameters**	
Calcium, mg/dL	9.5 [9.133-9.945]
Phosphorus, mg/dL	4.8 [4.183-5.643]
PTH, pg/mL	256 [134.85-492.413]
Albumin, mg/mL	3.4 [3.1-3.8]
**Follow up, months**	17.2 [10.2-28.4]
**Outcomes**	
Cause of death (cardiovascular), %	6.8
Death event, %	18.2

CAD: coronary artery disease; BMI: body mass index; PD: Peritoneal Dialysis; PTH: Parathormone.

**Figure 1:**
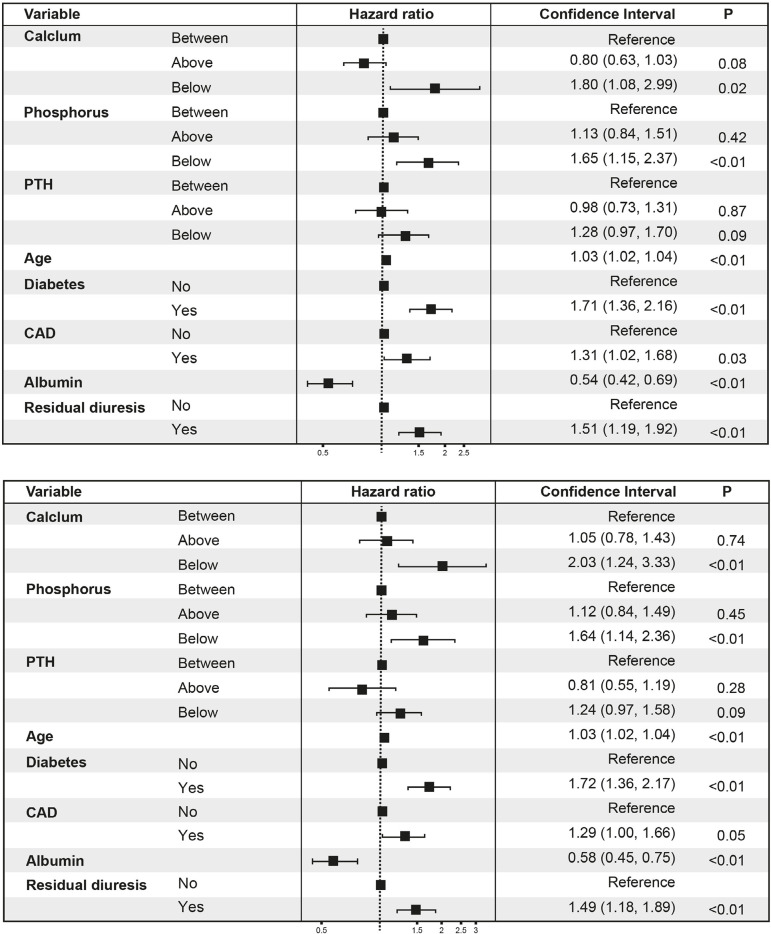
Groups analyses. HR for all causes of mortality from multivariable Cox models and log-rank test, comparing patients with values between versus below and above guideline ranges for calcium, phosphorus, and parathormone, according to KDOQI (left) and KDIGO (right).

We constructed two cox-models based on ranges of Ca, P, and PTH proposed by KDOQI and KDIGO (Ca: 8.4-9.5 mg/dL, P: 3.5-5.5 mg/dL, and PTH: 150-300 pg/mL proposed by KDOQI, and Ca: 8.4-10.2 mg/dL, P: 3.5-5.5 mg/dL and PTH: 150-600 pg/mL proposed by KDIGO). The analysis shows significance between all-causes of mortality and patients with Ca and P below the minimum values of both guidelines (KDOQI and KDIGO). The results were plotted in Figure 1.

**Figure 2:**
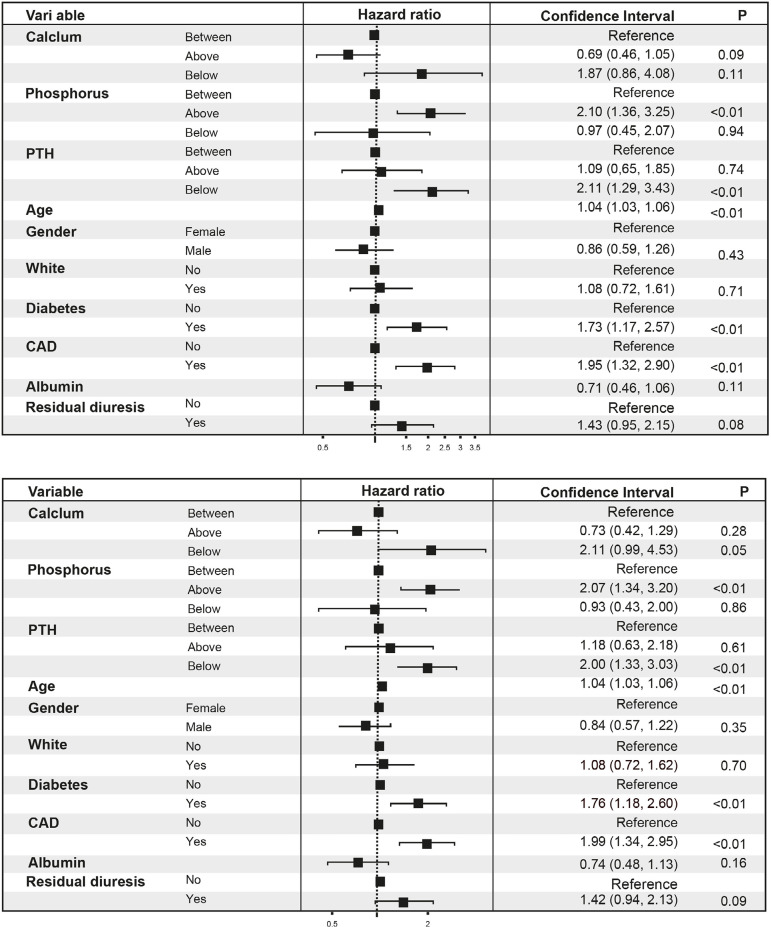
Group analyses. Hazard ratio for cardiovascular mortality from multivariable Cox models and log-rank test, comparing patients with values between versus below and above guideline values for calcium, phosphorus, and parathormone according to KDOQI (left) and KDIGO (right).

**Figure 3:**
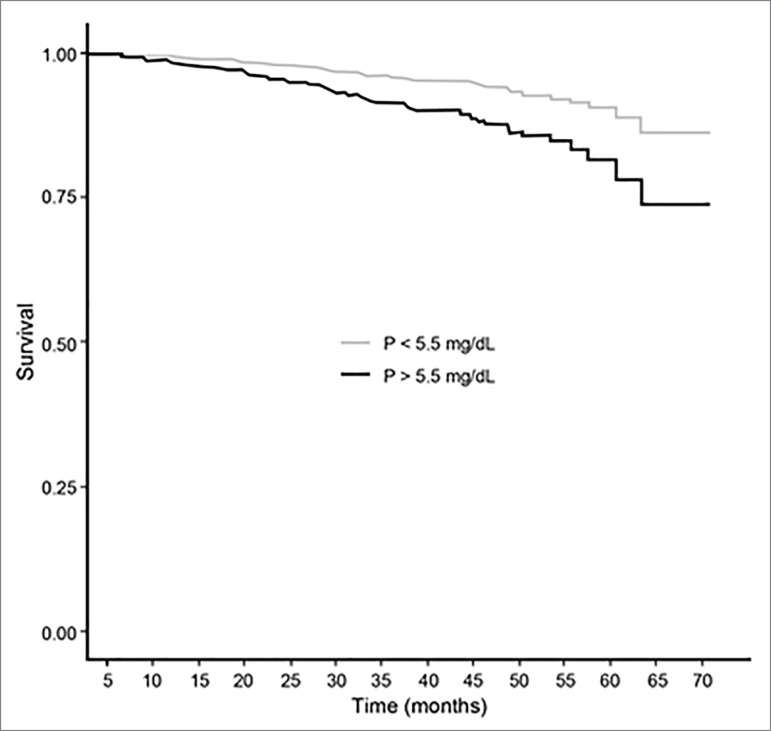
Cox regression survival curves for cardiovascular mortality of patients with phosphorus value below versus above 5.5 mg/dL (adjusted for: calcium, parathormone, age, gender, race, diabetes, coronary artery disease, albumin and residual diuresis). Hazard ratio: 2.08 (1.36-3.18), p-value: < 0.01. Log-rank test.

**Figure 4:**
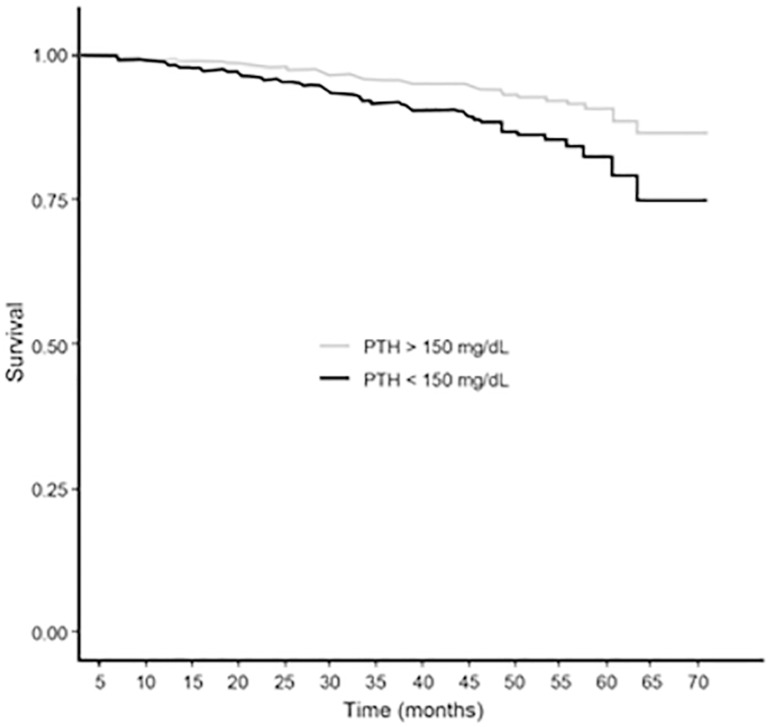
Cox regression survival curves for cardiovascular mortality of patients with parathormone below versus above 150 pg/mL (adjusted for: calcium, phosphorus, age, gender, race, diabetes, coronary artery disease, albumin and residual diuresis). Hazard ratio: 1.96 (1.33-2.90), p-value: < 0.01. Log-rank test.

We divided patients into cardiovascular and non-cardiovascular death, and the results are shown in Table 2. Gender, race, diabetes, CAD, RD, serum P, PTH, and albumin were associated with cardiovascular mortality by univariate analysis. To assess the association between Ca, P, and PTH (by KDOQI and KDIGO ranges) and cardiovascular mortality, two models were constructed and plotted in Figure 2. We found significantly increased cardiovascular mortality associated with serum P levels above and serum PTH levels below KDOQI and KDIGO ranges. Figures 3 and 4 show a significant difference between P and PTH levels (within or outside of KDOQI and KDIGO ranges) associated with cardiovascular mortality, and competing risk analysis confirmed these results taking into account other causes of death and censored patients (Table 3).

**Table 2:** Baseline characteristics of patients according to cardiovascular death

Variable	Non cardiovascular death	Cardiovascular death	p-value
n	4124	300	
Demographic parameters			
Age, years	59.2 [48.5 - 70.1]	68 [58.4-75.1]	< 0.01
Gender (female), %	53.3	47.7	0.06
Race (white), %	62.4	68.7	0.02
Hypertension (no), %	76.6	76.3	0.25
Diabetes (yes), %	43.6	58.3	< 0.01
CAD (no),%	20.6	37.3	< 0.01
BMI, Kg/m^2^	24.1 [21.6 - 27.2]	24.5 [22.1-28.1]	0.16
Residual diuresis (no), %	70.1	65.3	0.04
**Laboratory parameters**			
Calcium, mg/dL	9.5 [9.1-9.9]	9.6 [9.2-10.1]	0.32
Phosphorus, mg/dL	4.8 [4.2 - 5.6]	4.7 [4-5.5]	< 0.01
PTH, pg/mL	263 [138 - 493]	190 [102.9-428]	0.01
Albumin, mg/mL	3.5 [3.1 - 3.8]	3.4 [3-3.7]	0.09
**Follow up, months**	17.2 [10.2 - 28.4]	15 [9.6-25.4]	< 0.01
**Death, %**	12.3	100	

CAD: coronary artery disease; BMI: body mass index; PTH: Parathormone.

**Table 3:** Cardiovascular death as a competing risk

Variable	Coefficient (SE)	Subdistribution hazard ratio	p-value
Calcium	0.60	0.55	0.33
Phosphorus	0.22	2.17	< 0.01
PTH	0.20	1.76	< 0.01
Age	0.09	1.04	< 0.01
Diabetes	0.20	1.68	0.01
Residual diuresis	0.20	0.76	0.18
Albumin	0.25	0.99	0.98
CAD	0.21	2.00	< 0.01

Competing risk analysis for cardiovascular mortality of patients with calcium values below versus above 10.2 mg/dL, phosphorus below versus above 5.5 mg/dL, and PTH below versus above 150 pg/mL (adjusted for: age, gender, diabetes, residual diuresis , albumin, and CAD). Log-rank test. PTH: Parathormone; CAD: coronary artery disease.

## Discussion

MBD has an important impact on dialysis patients' morbidity and mortality. This is the first study attempting to analyze the impact of current recommended target levels of Ca, P, and PTH on cardiovascular mortality of PD patients. It was concluded that the levels of P above and PTH below those proposed by KDOQI and KDIGO dealing with MBD-CKD are associated with cardiovascular mortality in PD patients.

### MBD and mortality

The development of the KDOQI and KDIGO guidelines has contributed to improve the management of CKD-MBD. However, both HD and PD patients find it difficult to achieve the goals determined by these guidelines and complications are still present^10^^,^ 11. KDIGO cited several studies that showed the association between CKD-MBD and all-cause mortality and cardiovascular mortality, and most of them used Ca, P, and PTH as a marker of MBD^13^^-^ 16. The mechanism of vascular calcification is still unclear. However, the enrollment of high levels of Ca and P are taken for granted. High and low PTH levels are associated with vascular calcification, and low PTH levels can explain high mortality in these patients, especially if we consider that lower levels of PTH are more common in PD patients.

### Calcium

Several factors have been speculated to be triggers of vascular smooth muscle cell (VSMC) osteogenic differentiation. Osteogenic differentiation occurs when VSMCs are exposed to high levels of Ca, which explains the association between high levels of serum Ca and the presence of vascular calcification in CKD patients^17^^,^ 18. Publications that supported the KDOQI guideline did not analyze the Ca value alone as a determining factor, but considered the product Ca x P to define it. Until the years 2000 several studies have valued the Ca x P product, which should not exceed 55, at the risk of favoring vascular calcification and decreasing survival^19^. However, this type of calcification, common in CKD participants, is a complex and regulated process involving inhibitory and inductive molecules in addition to the differentiation of smooth muscle cells that assume the osteoblast phenotype promoting calcification^20^. This new knowledge diminished the importance attributed to the Ca x P product in the process of vascular calcification^21^^,^ 22. Studies with low risk of bias quoted by KDIGO found an association between low levels of Ca and mortality in line with our findings and opposing the theory of high VSMCs exposition to high Ca concentration as trigger of osteogenic differentiation^11^. Tentori et al. showed an association between low and high levels of Ca and mortality, and greatest mortality associated with high levels of Ca; this was the largest study (n = 25,529) cited by KDIGO^23^.

### Phosphorus

Hyperphosphatemia is the most important inducer of vascular calcification. Type III sodium-dependent Pi co-transporters (Pit-1) exposure to elevated P concentration activate signaling pathways and contribute to vascular calcification. Removal of P in PD is similar to patients on HD, approximately 2400 mg/week, done by diffusion and convection^24^^-^ 26. KDOQI and KDIGO proposed a similar range of P, and some studies have found association between this range and cardiovascular mortality, all of them in HD patients. Tentori et al. found the association in the largest study (n = 25,529), and Kimata et al. (n = 5,041) and Eddington et al. (n = 1203) have found similar associations between P and cardiovascular mortality also in HD patients, all of them categorized as medium risk of bias by KDIGO^23^^,^ 27 ^,^ 28.

### PTH

Evidence suggests that PTH may be an independent risk factor for cardiovascular mortality. A study with more than 40,000 participants on HD found an association between mortality and PTH levels above 600 pg/mL^25^. A study that followed 958 participants for a mean period of 9.7 years, with creatinine clearance around 62 ± 14 mL/min/1.73 m^2^, indicated PTH as a predictor of mortality due to cardiovascular causes^29^, based on the fact that elevated PTH acts on the myocardium inducing left ventricular hypertrophy, fibrosis, and vascular calcification^30^.

KDOQI did not evaluate survival due to PTH levels, but rather the values of this hormone which were able to discriminate the type of bone turnover were obtained from the histomorphometric analysis of bone biopsies, reaching the conclusion that this value was between 100 and 300 pg/mL^10^. Wang et al. analyzed bone biopsies from 175 HD participants demonstrating that PTH levels between 100 and 300 pg/mL separated participants with low and high turnover, which was also observed in the study by Solal et al.^31^^,^ 32.

Regarding PTH levels and mortality for KDIGO, studies such as the DOPPS that indicated values between 101 and 300 pg/mL, which had lower mortality and an expressive increase with PTH higher than 600 pg/mL, were taken as references^23^. The COSMOS study correlated the value of 398 pg/mL with the best survival, accepting a variation between 168 and 674 pg/mL^5^. Fouque et al. showed that PTH values between 100 and 1090 pg/mL showed better survival and finally, Floege et al. observed better survival at PTH levels between 75 and 600 pg/mL^14^^,^ 15. Barreto et al. evaluated 97 biopsies of HD participants, with a one-year interval, showing that those who remained with PTH levels within the values proposed by KDOQI had a high incidence of low bone turnover^33^. Based mainly on this study, KDIGO proposed other PTH values (between 2 and 9 times the value of the method - 150 to 600 pg/mL) as the ones that best discriminated bone turnover^11^.

We have shown an association between cardiovascular mortality and lower PTH levels, and our findings are in line with that of the COSMOS study, as well as Avram et al. and Liu et al. studies, who found an association between higher all-cause mortality rate and lower PTH levels^5^^,^ 34. Asci et al. found an association between CAC score and low bone turnover and age and DM in HD patients. In line with this study, we found an association between cardiovascular mortality and low levels of PTH and age and DM^35^.

### Limitations

This was a nationwide cohort with patients from different regions of Brazil, which differ in terms of social, economic, eating habits, and mixed-race factors and this may interfere with the results^36^.

Importantly, few publications were careful in selecting participants treated with PD for more than 6 months as we did. This was done to assess the effect of PD on MBD, reducing the interference of conservative management for participants with CKD. However, we can not completely exclude selection bias.

Although residual renal function is important for prognosis in patients treated with PD, as well as characteristics of membrane transport, KtV, and the doses of calcium carbonate intake and sevelamer, these variables were not available for the majority of participants and were not included in our analyses.

MBD affects quality of life and could cause various comorbidities (fractures, bone pain, increased hospitalization, and cardiovascular complications)^37^^-^ 42. Our study was limited to assessing the impact of these disorders on the survival of participants on PD. Other studies are needed to assess whether a better control of MBD affects morbidity and quality of life of PD participants.

## Conclusion

This is the first study to demonstrate that P above and PTH below the values proposed by KDOQI and KDIGO increase cardiovascular mortality in PD patients.
